# RNAseq Analysis Reveals Virus Diversity within Hawaiian Apiary Insect Communities

**DOI:** 10.3390/v11050397

**Published:** 2019-04-27

**Authors:** Laura E. Brettell, Declan C. Schroeder, Stephen J. Martin

**Affiliations:** 1 Hawkesbury Institute for the Environment, Western Sydney University; Locked bag 1797, Penrith 2751, NSW, Australia; 2 School of Environment and life Sciences, University of Salford, Manchester, M5 4WT, UK; s.j.martin@salford.ac.uk; 3 School of Biological Sciences, University of Reading, Reading RG6 6LA, UK; dcschroe@umn.edu; 4 Veterinary Population Medicine, College of Veterinary Medicine, University of Minnesota, St Paul, MN 55108, USA

**Keywords:** RNAseq, honey bees, deformed wing virus, quasispecies, apiary pests, recombination

## Abstract

Deformed wing virus (DWV) is the most abundant viral pathogen of honey bees and has been associated with large-scale colony losses. DWV and other bee-associated RNA viruses are generalists capable of infecting diverse hosts. Here, we used RNAseq analysis to test the hypothesis that due to the frequency of interactions, a range of apiary pest species would become infected with DWV and/or other honey bee-associated viruses. We confirmed that DWV-A was the most prevalent virus in the apiary, with genetically similar sequences circulating in the apiary pests, suggesting frequent inter-species transmission. In addition, different proportions of the three DWV master variants as indicated by BLAST analysis and genome coverage plots revealed interesting DWV-species groupings. We also observed that new genomic recombinants were formed by the DWV master variants, which are likely adapted to replicate in different host species. Species groupings also applied when considering other viruses, many of which were widespread in the apiaries. In social wasps, samples were grouped further by site, which potentially also influenced viral load. Thus, the apiary invertebrate community has the potential to act as reservoirs of honey bee-associated viruses, highlighting the importance of considering the wider community in the apiary when considering honey bee health.

## 1. Introduction

Across the globe, emerging infectious diseases (EIDs) pose a significant threat to biodiversity and health [1]. This has been clearly demonstrated in recent years both by the recent catastrophic decline of amphibians caused by pathogenic fungi [2] and by the cases of large-scale honey bee colony losses, a major factor in which is the spread of pathogenic viruses [3].

EIDs often occur as a consequence of human-mediated translocations of infected hosts and/or parasites and due to the close proximity of wild and domesticated hosts [1]. As such the honey bee, which over the last century has been spread across the globe by humans for honey production and pollination services [4] and shares complex communities with a wide range of insect taxa [5,6,7,8], is a prime candidate to facilitate the spread of EIDs into new insect hosts. Pollinators and other insects with which they share environments are of particular interest due to their value in terms of economy (including pollination services) and biodiversity and are currently already experiencing a number of pressures, e.g., from habitat loss and pesticides [9,10,11]. The combination of multiple pressures can have the additional effect of decreasing immunity and thus potentially increasing susceptibility to pathogens [12], although further studies are needed in this area [13].

Most host switching results in a dead end or a limited low-level outbreak; however, on rare occasions the transmission can result in sustained outbreaks or major epidemics. This can happen when there is sufficient increased exposure or the evolution of new variants in the original or new host allows successful replication and efficient spread between members of the new host species [14]. RNA viruses with their high mutation rates, diverse sequences, and often very high population sizes [15] are thus prime candidates for emergence or re-emergence in novel hosts. This is of particular concern when considering honey bee populations, which host abundant and diverse RNA viruses, are frequently transported en masse, and come into frequent contact with other arthropods both in the hive and when foraging.

Deformed wing virus (DWV) is now well known to be one of the major factors responsible for honey bee colony losses across the world [3,4,16]. This single virus has come to dominate the virome of honey bee populations due to its spread and amplification in the host being aided by the ectoparasitic mite *Varroa destructor* (referred to as varroa from now on) [17,18]. Additionally, the new mite–bee transmission route serves to reduce viral genotypic diversity and select for the amplification of virulent strains [16,17]. Although initially described as a honey bee virus, it has since become apparent that DWV is a generalist insect virus capable of infecting 64 species from eight orders of arthropods [19]. However, the extent of the generality and capacity for emergence in new hosts is still the subject of contention [20]. Worryingly, recent studies have found that viral pathogens circulating in managed pollinators may be driving infections in wild species [21,22], and in the Hawaiian system, it has recently been shown using RT-PCR-based studies that the presence of varroa in honey bee populations has resulted in a dramatic increase in the prevalence of DWV in species of wasps (*Polistes* sp.) and solitary bees (*Ceratina smaragdula*) [23]. Furthermore, in the yellowjacket wasp (*Vespula pensylvanica*) on the Big Island, Hawaii, DWV prevalence has increased along with a decrease in strain diversity [24], mirroring the situation seen in honey bees [17]. 

Although gene targeted RT-PCR and RT-qPCR have provided very useful insights into the virome of insect communities, RNAseq allows entire genomes of RNA viruses to be sequenced easily and thus provides much deeper anaylsis. More specifically, using oligo dT-derived RNAseq on field collected samples in Hawaiian honey bee apiaries in which varroa is established, we aimed to identify the extent to which +ssRNA viruses (the overwhelming majority of viruses found associated with honey bees are +ss polyadenylated RNA viruses [10]) were present in the wider arthropod community and to characterize any DWV master variant genomes present in the different species. Common pest species were targeted as they would have the most frequent contact with honey bees that are known to harbor high DWV loads in the study locations [17,25]. We hypothesized that the frequency of interactions would result in common DWV genotypes circling in the apiary environment due to repeated viral transmission events between species. Furthermore, we aimed to determine whether other viruses commonly found in honey bees are also found in apiary pests, and if so, whether certain viruses are associated with particular hosts. The pest species investigated were varroa mites (*Varroa destuctor*), small hive beetles (*Aethina tumida*) yellowjacket wasps (*Vespula pensylvanica*), and two species of ant, big headed ants (*Pheidole megacephala*) and ghost ants (*Tapinoma melanocephalum*)—all widely distributed invasive pests known to interact directly with honey bees in Hawaii.

## 2. Materials and Methods

### 2.1. Site and Species Selection

**Figure 1:**
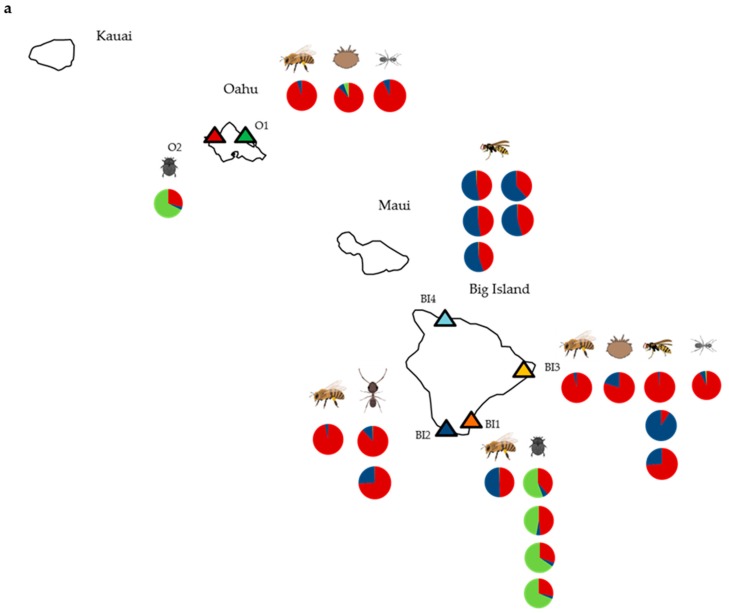

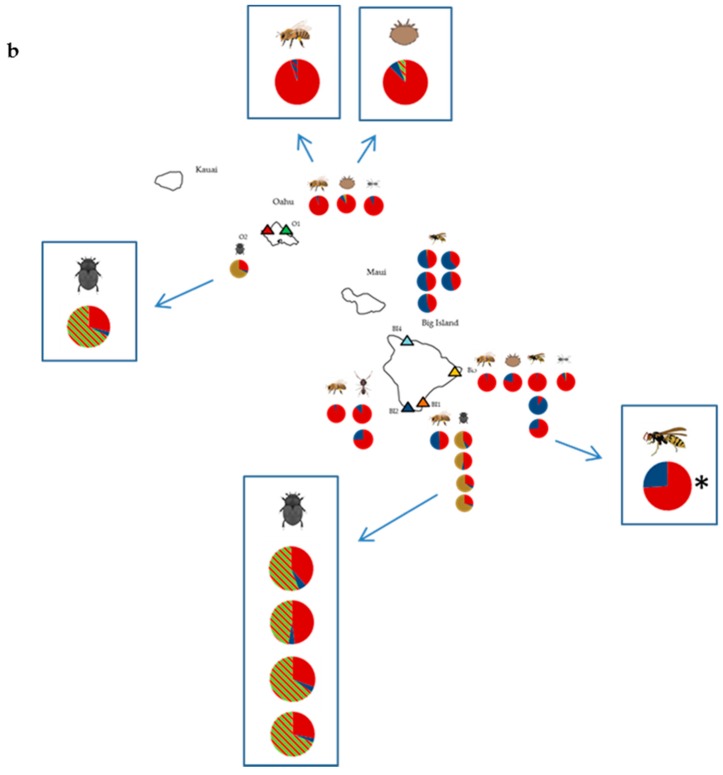
Proportions of deformed wing virus (DWV)-like reads in individual samples from each species mapping to DWV type A (red), type B (blue), and type C (green) along with the site from which they were collected. (**a**) Results using BLAST top hit analysis only (read numbers provided in Table S4); (**b**) where results were amended after considering additional Geneious mapping analysis (Figure S1). Green and red stripes represent that DWV-A/DWV-C recombinants were present (rather that true full-length DWV-C,), red and blue stripes denote a DWV-A/DWV-B recombinant (no full-length DWV-B), and the asterisk represents an additional sample identified as containing recombinant(s). Results for all other samples remain unchanged. Insect images are from BioRender [23,31].

In November and December 2012, opportunistic sampling was carried out in managed apiaries on the islands of Oahu and the Big Island, Hawaii (Figure 1), and where found, common apiary pests (yellowjacket wasps, small hive beetles, big headed ants, and ghost ants) were collected from within brood boxes or at hive entrances. Additionally, reference samples of asymptomatic honey bees (50–100 individuals per hive) were also collected. Varroa populations had been established for at least 3–5 years at all locations sampled, and honey bee populations were known to harbor high DWV levels [17,25]. All samples were kept on ice for transportation to the laboratory, where they were stored at –80 °C. Additional samples of small hive beetles had been collected in the same way earlier in the year from colonies in an advanced stage of collapse, i.e., “slime out phase”.

### 2.2. RNA Extraction and Next Generation Sequencing

Pools of 30 asymptomatic honey bees were taken and checked for the presence of varroa mites, which, where found, were removed and stored separately. The pools of whole bees were then ground in liquid nitrogen using a sterile pestle and mortar to produce a fine homogenous powder, 30 mg of which was used for RNA extraction. Varroa mites that had been removed from bees were homogenized in pools of 10 using a mini pestle in a 1.5 mL eppendorf tube. Small hive beetles were homogenized either individually or in pools of six. Yellowjacket wasps were homogenized individually, and all ants were crushed in pools of 20–40 due to their small size. RNA extractions proceeded for all samples in the same way using the RNeasy mini kit (Qiagen, Manchetser, UK) following the manufacturers’ instructions, eluting in 30 μL nuclease free water followed by DNase treatment using DNase I (Promega, Southampton, UK). Initial screening for the presence of DWV was then carried out using RT-PCR as per [17,26]. A selection of positive samples was then chosen to be analyzed in greater depth using total RNA sequencing (RNAseq). The list of samples used for RNAseq analysis along with the species name, site from which they were sampled, and symbol used to denote them in subsequent figures is given in Table S1. These samples were transported to The BBSRC Earlham Institute (Norwich), where cDNA libraries were prepared using oligo (dT) priming. Resulting libraries were then run on the HiSeq 2000 (The Earlham Institute, Norwich). Raw data were deposited in the National Centre for Biotechnology Information (NCBI) Sequence Read Archive (SRA) under BioProject accession number: PRJNA531527. Sample V_des_2 was previously deposited in the European Nucleotide Archive (ENA) as sample “V_S48” under the Study Accession PRJEB8112.

### 2.3. Bioinformatic Analysis

Initially, quality control of generated reads was performed using FASTQC (http://www.bioinformatics.babraham.ac.uk/projects/fastqc/). Then, a pipeline initially described by [25] was applied to identify DWV-like reads in individual samples. Briefly, this involved taking all reads that passed QC and performing a nucleotide BLAST against a custom database (*E* value of 10^−5^) containing DWV types A, B. and C (type A: NC_004830.2 and NC_005876.1 (Kakugo virus), type B: AY251269.2 (VDV-1), and type C: CEND01000001.1). Custom perl, sed, and awk scripts were used to parse the data, take the top BLAST hit, remove empty lines, and remove reads which did not map to the database. BLAST top hit analysis was then used to quantify the numbers of reads belonging to each of the three master variants by identifying which of the reference genomes each read matched to most closely. This was used to produce pie charts showing the proportions of DWV type A, B, and C reads in each sample.

To assemble contigs representing the full diversity of DWV sequences present in each sample, DWV-like reads were identified by BLAST (read1 files for each sample in fasta format), and the corresponding read 2 files were selected. These were then used to generate de novo assemblies using VICUNA, an assembler specifically designed to accommodate the highly variable sequence data typical of RNA viruses [27].

To investigate DWV diversity within the apiary insect community, all VICUNA-generated contigs >300 bp for each sample were imported into Geneious (Version 7.04, Biomatters), and the map to reference tool was then used with a MUSCLE alignment to competitively map contigs to the DWV-A (NC_004830.2), -B (AY251269.2), and -C (CEND01000001.1) reference genomes (mapping ambiguous reads to all). The resulting alignments containing all the DWV contigs were then trimmed to use a 507 bp region of the *RdRp* gene (nucleotide positions 8016–8522 on NC_004830.2, 7989–8495 on AY251269.2, and 7999–8505 on CEND01000001.1 [4]). Alignments were visually inspected, and contigs showing recombination breakpoints were removed so as to comply with the assumptions of the phylogenetic construction, and a Baysian phylogenetic analysis was constructed using the MrBayes v3.2.6 plugin in Geneious. [28], using a GTR substitution model with gamma rate variation. We ran four chains for 1.1 × 10^6^ MCMC generations, sampling trees every 200 generations, and a consensus tree was then created using a 10% burn in and 50% support threshold.

Furthermore, to understand the diversity of DWV-A in Hawaiian apiaries in a global context, a second phylogeny was generated using a 410 bp region of the *RdRp* gene, as used by [4] in their global DWV-A phylogeny along with 10 of their DWV-A sequences of geographically diverse origins from both honey bees and varroa mites (Table S2). DWV-A contigs generated in this study were aligned with the 410 bp sequences from [4] in Geneious (MUSCLE alignment), and this time, no recombinant contigs were observed in the alignment. A Bayesian phylogeny was then created using the same parameters as described above. The corresponding region of the DWV-C reference genome (CEND01000001.1) was used as the outgroup.

To determine whether recombinants were dominating in the samples, competitive alignment plots were created to look at the DWV reference genomes to which reads preferentially mapped across the length of the genome. This used all reads passing QC (read 1, in fasta format) and the “Map to Reference” tool in Geneious, using DWV types A, B and C (NC_004830.2, AY251269.2 and CEND01000001.1) reference genomes, discarding all ambiguous reads. Recombinants were observed to be present if preferential coverage switched from one master variant to another along the length of the genome. To provide additional evidence for the presence of recombinants, two samples were chosen for which competitive alignment plots had revealed recombinants to be present (V_pen_8 and A_tum_5), and their de novo assembled DWV contigs were aligned using MUSCLE and visually inspected to identify recombinant contigs.

Finally, to investigate whether other honey bee-associated viruses were circulating in the apiary community, a custom BLAST database was created using DWV types A, B, and C along with seven common honey bee-associated viruses and an additional two viruses (Moku and Milolii viruses [29,30]) previously identified from RNAseq data generated from Hawaiian apiary insects (data from individual insects were also used in this study) (Table S3), and BLASTn was used to identify reads belonging to each virus (*E* value 10^−5^). Read counts were expressed as reads per kilobase million (RPKM). 

### 2.4. Statistical Analysis

Redundancy analysis (RDA) was carried out in R (version 3.3.2) to investigate whether the response variables of read count data (RPKM) for each virus were associated with explanatory variables of insect species and site. Dummy variables were randomly assigned as varroa for the species category and B4 for site.

## 3. Results

### 3.1. Deformed Wing Virus

RNAseq data was generated from samples of honey bees (A_mel_1-A_mel_4), small hive beetles (A_tum_1-A_tum_5), yellowjacket wasps (V_pen_1-V_pen_8), big headed ants (P_meg_1 and P_meg_2) and ghost ants (T_mel_1 and T_mel_2). As expected from our previous work [17,25], BLAST top hit analysis revealed DWV to dominate sequence reads in all honey bee and varroa mite samples, reaching as high as 91.25% of total reads in the varroa sample V_des_2 from apiary O1. DWV was ubiquitous, being detected in all ant, wasp and beetle samples, although read counts were highly variable (Table S4). The proportions of DWV-types A, B and C present also differed considerably and appeared to group by species (Figure 1). Of the DWV reads, DWV-A reads dominated the majority of honey bee, varroa and ant samples; however the small hive beetle samples were all dominated by type C and the wasps usually contained relatively similar proportions of types A and B. Interestingly, the wasp samples showed further separation by location.

**Figure 2:**
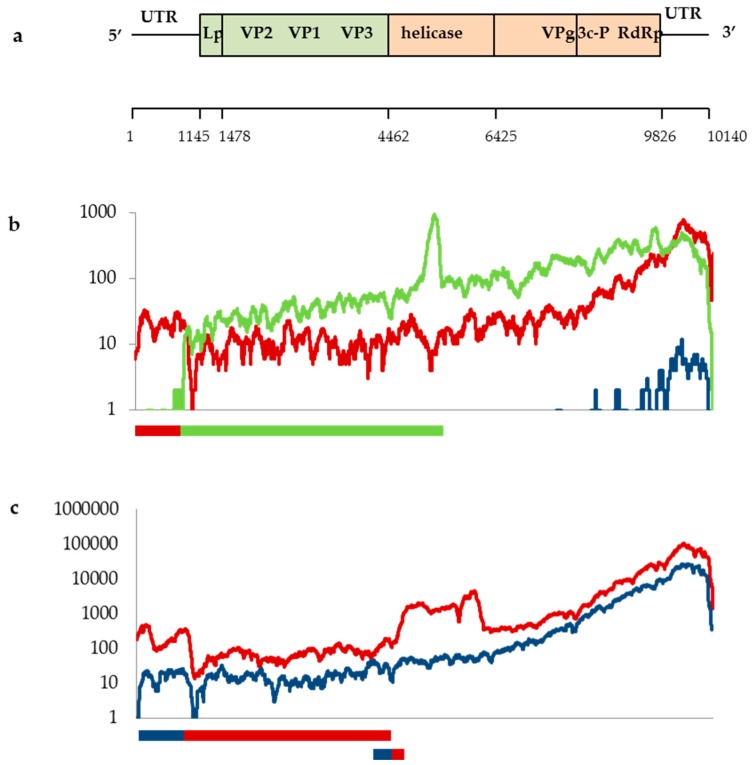
DWV genome coverage plots showing read depth of DWV-A (red), DWV-B (blue), and DWV-C (green), across the length of the genome, along with representations of VICUNA-assembled contigs showing recombination breakpoints; (**a**) genome organization with the structural region in green and the non-structural region in orange, showing nucleotide positions below (adapted from [33,34]); (**b**) small hive beetle sample A_tum_5; (**c**) yellowjacket wasp sample V_pen_8. Plots for all samples are shown in Figure S1, and nucleotide alignments showing the recombinant contigs for samples A_tum_5 and V_pens_8 are shown in Figure S2.

DWV genome coverage plots were generated for each sample, and all alignments showed the typical 3’ bias resulting from the oligo (dT) priming method of cDNA synthesis in library preparation [32]. Similarly, the samples containing type C all showed a spike in the Helicase region caused by the presence of a poly-(A) region of the genome, irrespective of location (Figure 2; Figure S1). Plots showed that the majority of samples were dominated by either full-length DWV-A or DWV-B sequences (or both), as demonstrated by the consistent coverage depth across the genome, and were consistent with the results of the BLAST top hit analysis (Figure 1a). Again, the yellowjacket wasp data appeared to separate by site, with samples V_pen_1–V_pen_5 from site B4 all showing identical coverage plots, with coverage being restricted to the 3’ end indicative of low virus levels in the samples and showing almost identical coverage for DWV-A and DWV-B. The samples V_pens_6 to V_pen_8 from site B3, however, were more variable and contained greater coverage depths, again, in keeping with the BLAST top hit analysis. 

Although the majority of samples were dominated by full-length genomes, recombinants were detected in this study as evidenced by the competitive alignment plots (Figure 2) and associated alignments of assembled contigs (Figure S1). The dominant master variants present were the same using both BLASTn and mapping to DWV reference genomes (Geneious). However, the mapping (competitive alignment plots) revealed additional information regarding the presence of recombinants. These additional data were therefore used to create amended pie charts showing variant proportions (Figure 1b) for samples where recombinants were detected. Interestingly all beetles were dominated by a DWV-A/DWV-C recombinant (Figure 1b, Figure 2b, Figures S1 and S2) with a breakpoint in the 5’ UTR immediately upstream of the open reading frame, albeit at low levels, irrespective of location. These beetles also contained lower level full-length DWV-A, as evidenced by type A coverage across the whole genome. The same recombinant was also present in the honey bee sample A_mel_4 (Figure S1), which was collected from a different location from any of the beetles, although the recombinant did not dominate in the honey bee sample, which was instead dominated by full-length DWV-A. Finally, one yellowjacket wasp sample, V_pen_8, showed a distinct coverage pattern not seen in any other sample in this study (Figure 2c). This sample showed three recombination breakpoints—one in the 5’ UTR and one at either end of the helicase gene—and showed coverage of both DWV-A and DWV-B across the full length of the genome. As such, these data cannot confirm the makeup or proportions of recombinant(s) and full-length genomes in this sample.

**Figure 3:**
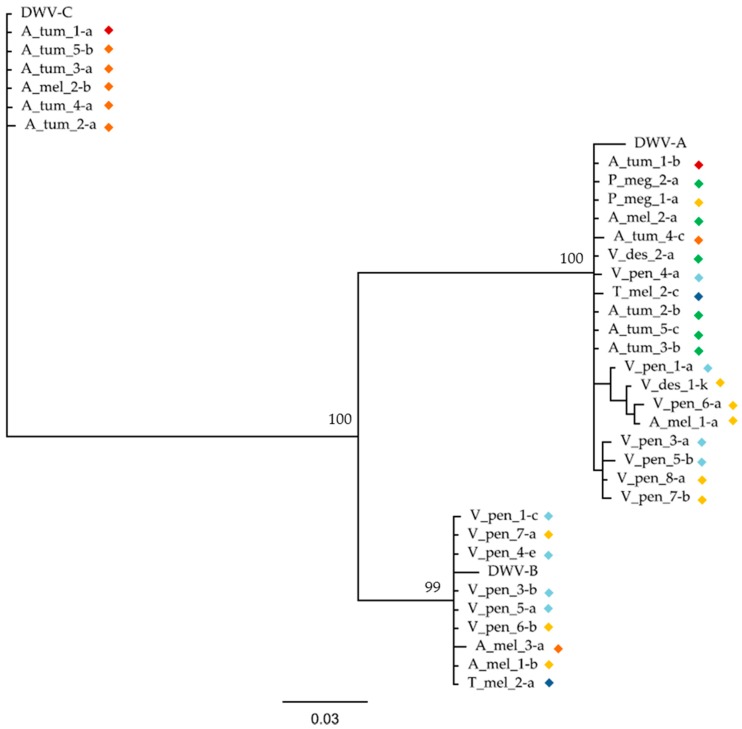
A Bayesian phylogeny of a 507 bp region of the DWV *RdRp* gene using trimmed contigs assembled for each sample individually using VICUNA, along with the corresponding regions from the DWV-A, B, and C reference genomes (NC_004830.2, AY251269.2, and CEND01000001.1). Colored diamonds represent the sample collection location: O1 = green, O2 = red, B1 = orange, B2 = dark blue, B3 = yellow, and B4 = light blue. Sample name suffixes represent the contig name. The bar represents the number of nucleotide substitutions per site, and the consensus support (%) is shown for selected branches.

A Bayesian phylogeny created using a 507 bp fragment of the *RdRp* gene assembled from all samples showed sequences to cluster according to master variant (DWV-A, B, and C), as expected (Figure 3). The origin of all the DWV-C sequences came from a single DWV-C variant, as all the sequences obtained from this region were almost identical to one another and to the reference genome. Within the DWV-A and DWV-B clades, samples did not cluster by either species or location and showed often very similar sequences, indicating that common variants are circling in the apiary.

**Figure 4:**
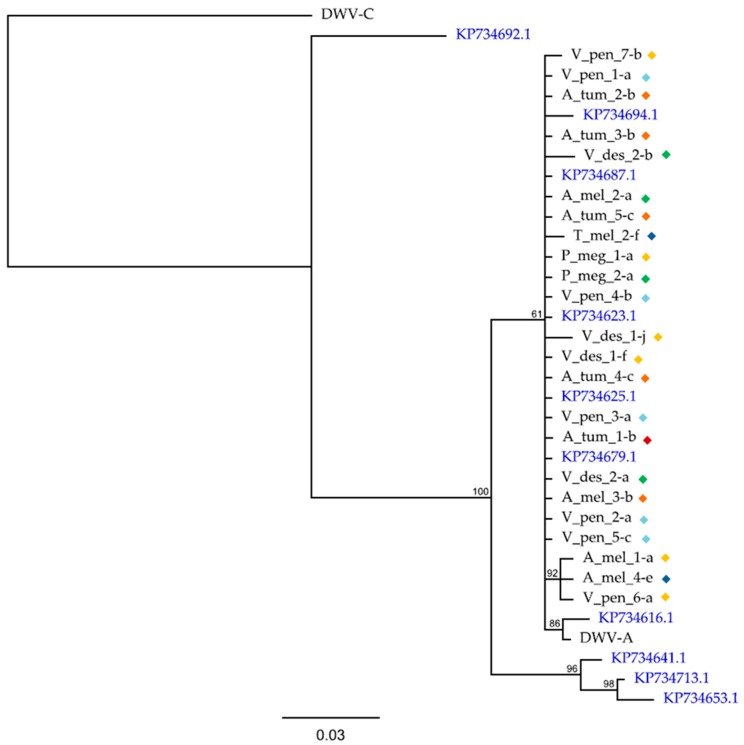
A Bayesian phylogeny of a 410 bp region of the DWV-A *RdRp* gene using trimmed contigs assembled for each sample individually using VICUNA, along with 10 additional sequences, as featured in the global DWV-A phylogeny of [4] (shown in blue text). Colored diamonds represent the site from which the sample was collected as follows: O1 = green, O2 = red, B1 = orange, B2 = dark blue, B3 = yellow, and B4 = light blue. Sample name suffixes represent the contig name. DWV-C is used as the outgroup, and the bar represents the number of nucleotide substitutions per site. Consensus support values (%) are shown.

To investigate how the DWV-A sequences in the Hawaiian apiary insects compared with DWV-A sequences on a broader scale, we constructed a phylogeny using the same 410 bp *RdRp* region used by [4] in their global phylogeny, along with some of their samples (Figure 4). This revealed that all samples in the current study fell within those detected elsewhere and clustered together with little sequence variation in this region of the genome.

### 3.2. Other Honey Bee-Associated Viruses

**Figure 5:**
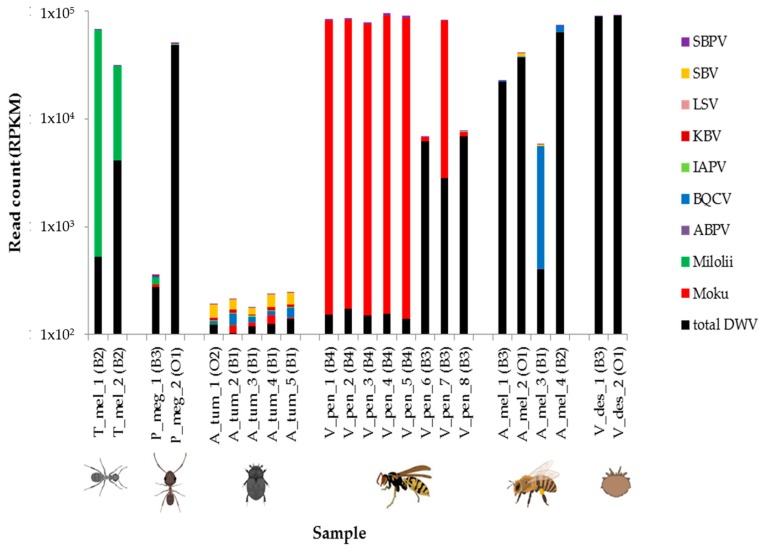
Histograms represent numbers of reads mapping to each of 10 commonly found insect viruses (slow bee paralysis virus: SBPV, sacbrood virus; SBV, Lake Sinai virus; LSV, Kashmir bee virus; KBV, Israeli acute paralysis virus; IAPV, black queen cell virus; BQCV, acute bee paralysis virus; ABPV, Milolii virus, Moku virus and deformed wing virus; DWV) as determined using BLASTn. Read numbers are expressed as reads per kilobase million (RPKM) and are shown on a log-10 scale starting 1 × 10^2^. The samples are positioned according to taxa: ghost ants (T_mel_1–2), big headed ants (P_meg_1–2), small hive beetles (A_tum_1–5), yellowjacket wasps (V_pens_1–8), honey bees (A_mel_1–4), and varroa (V_des_1–2). Apiary locations from where the samples were taken are given after each sample name.

In addition to generally grouping in terms of DWV types A, B, and C proportions and coverage, geographical and taxa groupings were also observed when considering the other honey bee-associated viruses they harbored. BLASTn was used to identify reads belonging to eight of the most common honey bee viruses and two additional viruses known from our previous work to be prevalent in Hawaiian insects. As expected, honey bees (with the exception of sample A_mel_3) and varroa were dominated by DWV reads, as were big headed ants. We found ghost ants to be dominated by Milolii virus, yellowjacket wasps to be dominated by Moku virus, and small hive beetles to contain relatively few virus reads (Figure 5). The wasp samples further separated into two groups, with samples V_pen_1–V_pen_5 (from site B4) containing high numbers of Moku virus reads and also consistent amounts of SBPV, whereas V_pen_6–V_pen_8 (from site B3) showed increased DWV, more variable Moku virus, and no notable SBPV. The honey bees were the only samples to contain any notable BQCV.

**Figure 6:**
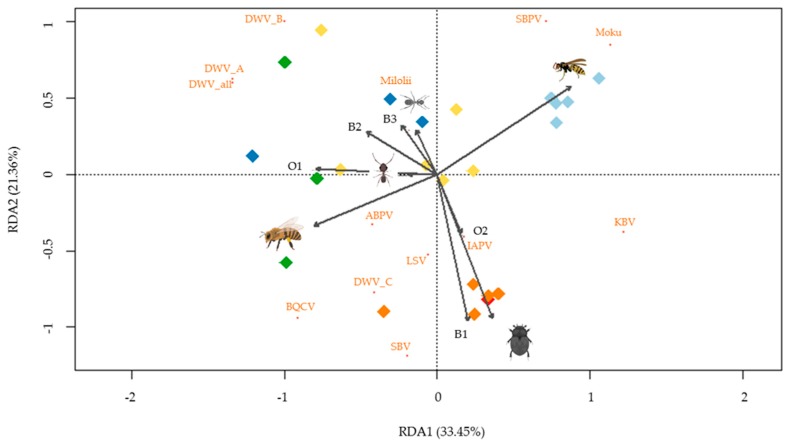
Redundancy Analysis (RDA) plot showing the variation in virus read count data (RPKM log-10) for each sample correlated with environmental variables: site and species. Dummy variables were randomly assigned to varroa (species) and B4 (site). Sites are colored: O1 = green, O2 = red, B1 = orange, B2 = dark blue, B3 = yellow, and B4 = light blue.

Redundancy analysis was conducted (*p* < 0.001) to further investigate the variation in viral read count data (log-10) for each sample in the context of species and site explanatory variables. RDA1 and RDA2 together explained 54.8% of the variation, and all variables (sites and species) were significantly different from the dummy variables (*p* < 0.05) (Figure 6). The RDA plot shows groupings by site (colors), as well as demonstrating separation by species, especially “honey bee”, “small hive beetle”, and “yellowjacket wasp”.

## 4. Discussion

This study revealed that common apiary pests have the potential to act as reservoirs for, or could be impacted by, a number of honey bee-associated viruses. Although recent studies have highlighted the ability of a number of viruses initially described as honey bee pathogens to infect a range of taxa and in some cases cause pathogenic effects (reviewed by [19]), this is the first study to assess the viral burden of taxonomically diverse common apiary pests.

Of 10 common +ssRNA viruses surveyed, DWV was the most common virus in honey bees and varroa, as expected, because it is the most prevalent virus in honey bees across the world [19] and was the most prevalent virus in the apiary with full-genome coverage achieved from samples of each species. DWV-A was the most common variant detected, although DWV-B was also widespread, correlating with the recent finding by [35] that DWV-B dominance is replacing DWV-A on the mainland United States of America (USA). Although the BLAST top hit analysis was reliable when considering full-length virus genomes, such as when detecting a number of different viruses (Figure 5), or when only full length master variants are present, e.g., considering DWV diversity in all ant samples (Figure 1 and Figure S2), methods that identify all reads independently, irrespective of genome location, mean that it is impossible to discriminate between full genomes and recombinant forms. This is evidenced by the beetle data that BLAST top hit analysis suggested was dominated by DWV-C; however closer inspection using competitive alignments revealed this to, in fact, be a DWV-A/DWV-C recombinant. Thus, the two analyses (BLAST top hit results and competitive alignment plots) gave consistent results when only full-length genomes were present but differed when recombinants were detected. 

Interestingly, beetles sampled from across two locations were the only ones to be dominated by a DWV-A/DWV-C recombinant. These all had very low read counts but were very consistent. All beetle samples were collected earlier in the year from collapsing honey bee colonies suffering from extremely heavy small hive beetle infestations (slime out conditions), and unfortunately, no corresponding bee samples were taken at this time. As such it may be possible that the same recombinant was present at high levels in the dying bees at that time, and the high amounts of virus in the hives resulted in passive contamination of the beetles, which would explain why DWV is only very rarely detected in beetles [36]. In this case, contamination during library preparation or during the Illumina run cannot be ruled out, because it is a particular issue for RNAseq analysis. This can occur with invertebrate viruses due to the extreme dominance of viral reads in heavily infected samples. These beetle samples had previously tested positive for DWV using RT-PCR prior to selection for RNAseq analysis and a recent RT-PCR-based study showed that stingless bees (*Melipona subnitida*) from Brazil also harbored DWV-C but at higher viral levels [37], confirming this DWV variant also has the ability to infect diverse hosts.

Investigating viral reads generated by oligo (dT), RNAseq is not the only method to assess whether samples are harboring true infections [38,39]. Nonetheless, this method will reveal high read counts, full-genome coverage, and assembled full genomes, suggesting that DWV is replicating in at least some samples from each species. Furthermore, the unique dominance of recombinant(s) in the wasp sample V_pens_8 that was not seen in any other sample provides further evidence that there are specific DWV variants/recombinants present in the quasispecies that are able to successfully replicate in different species. 

Considering both the phylogenies together, it appears that common DWV variants are circling in the apiary insect community, suggesting frequent interspecies transmission events. The nature of these transmission events remains unclear, with trophylaxis (between small hive beetles) [20], fecal–oral routes and predation on bees, and consumption of contaminated hive materials, pollen, and nectar all being implicated as routes by which viruses can spread both between and within species [40].

The DWV-A phylogeny, which in the original publication by [4] only contained honey bee- and varroa-derived sequences (Figure 5) showed all sequences from this study to fall within the greater diversity and all cluster within one clade. When considering the phylogeny of DWV sequences from the current study only (Figure 4), again all DWV-A clustered together as was also true of DWV-B sequences. The limitations both in terms of sampling and in using only one gene region means that is not possible to draw strong conclusions on the evolutionary history of these sequences. Furthermore, when considering the read count data and genome coverage plots for wasps only, they appear to separate by location with samples V_pen_1–5 from B4 all showing almost identical results in terms of their DWV variant composition, whereas V_pen_6–8 are very different (Figure 1 and Figure S1). These findings are in keeping with the results of [24] who found, using RT-PCR and Sanger sequencing, that DWV sequences in yellowjacket wasps (also from Hawaii) tracked strain changes observed in the honey bees [17] on which they were feeding, i.e., DWV diversity decreased in honey bees as varroa became established on Oahu and then on the Big Island, and this change was detected in yellowjackets on the Big Island. Although the current study is limited to eight individuals across two locations, it is clear that DWV is present in the wasp samples, that sequences are diverse, and that the presence and potential level of DWV variants are affected by location.

This is the first study of arthropods in the wider apiary community to consider the newly described Moku [29] and Milolii [30] viruses. Although these viruses only dominated in the samples in which they were first described, low numbers of reads were also found in other varroa, beetle, wasp, and ant samples. The wider +ssRNA virus detection study revealed that all of the common honey bee-associated viruses tested for were present at some level in the Hawaiian apiary insects, although IAPV, KBV, ABPV, and LSV were only present in very low levels (as detected through BLAST), and therefore, true infections cannot be confirmed in any sample. It is interesting to note that, similar to the findings of the DWV data, when considering the other honey bee-associated virus data, species and location groupings were seen. In the case of species groupings, this may be related to different species having different virus susceptibilities and, in the case of site groupings, may be more related to variation in particular virus levels at different sites.

## 5. Conclusions

Although this pilot study has limitations, namely unbalanced sampling design, we have shown that several common honey bee-associated +ssRNA viruses are common in taxonomically diverse apiary pests. We showed that DWV was the most prevalent virus and that DWV infections grouped between species in terms of dominant variants and recombinants, but that common variants (predominantly type A) were circling between all species, suggesting repeated transmission events between species. Within the wasps, DWV was further separated by location. Species also grouped in terms of which other honey bee-associated viruses they harbored, i.e., particular viruses are associated with particular hosts. Therefore, this study highlights the need to consider the wider arthropod community as potential reservoirs of viral pathogens in the apiary. 

## Supplementary Materials

The following are available online at https://www.mdpi.com/1999-4915/11/5/397/s1, Figure S1: DWV genome coverage plots for individual samples created using Geneious. Read depths are shown on a log-10 scale and represent DWV-A (red), -B (blue), and -C(yellow) along the ~10.1 kb genomes. Figure S2: DWV alignments (MUSCLE) created using Geneious showing de novo assembled contigs from samples A_tum-5 and V_pens-8, which contain recombination breakpoints. (a) Contig A_tum-5-a aligned with DWV-A (NC_004830.2) and DWV-C (CEND01000001.1) reference genomes, (b) contig V_pen_8-b aligned with DWV-A (NC_004830.2) and DWV-B (AY251269.2), and (c) a second contig from sample V_pen_8; V_pen_8-t also aligned with DWV-A (NC_004830.2) and DWV-B (AY251269.2). All alignments show disagreements with the assembled contigs highlighted in black, and recombinant contigs are shaded red where they map most closely to DWV-A, blue to DWV-B, and green to DWV-C. Table S1: Samples used in this study. Sample names are given along with the site from which they were sampled, species name, and the symbol used to denote them in Figure 1 and Figure 5. Table S2: DWV-A *RdRp* sequences originally from [4] and used in this study in the construction of the DWV-A phylogeny in Figure 5. Table S3: Viruses commonly found in bees used for BLAST analysis along with accession numbers. Table S4: Numbers of reads mapping to DWV types A, B, and C using Blast top hit analysis for each sample, along with total numbers of reads passing QC (read1.fasta).
