# Supplementary material for: RNAseq Analysis Reveals Virus Diversity within Hawaiian Apiary Insect Communities
Source: Viruses. 2019 Apr 27;11(5):397. doi: 10.3390/v11050397 (PMC6563275; doi:10.3390/v11050397)
Supplement: Supplementary file 1 [file viruses-11-00397-s001.pdf]

Supplementary Materials: The following are available online at www.mdpi.com/xxx/s1, Figure S1: DWV genome coverage plots for individual samples created using Geneious. Read depths are shown on a log-10 scale and represent DWV-A (red), -B (blue), and -C(yellow) along the ~10.1 kb genomes. Table S1: DWV-A *RdRp* sequences originally from [4] and used in this study in the construction of the DWV-A phylogeny in Figure 5. Table S2: Viruses commonly found in bees used for BLAST analysis along with accession numbers. Table S3: Numbers of reads mapping to DWV types A, B, and C using BLAST top hit analysis for each sample, along with location and total numbers of reads passing QC (read1.fasta).

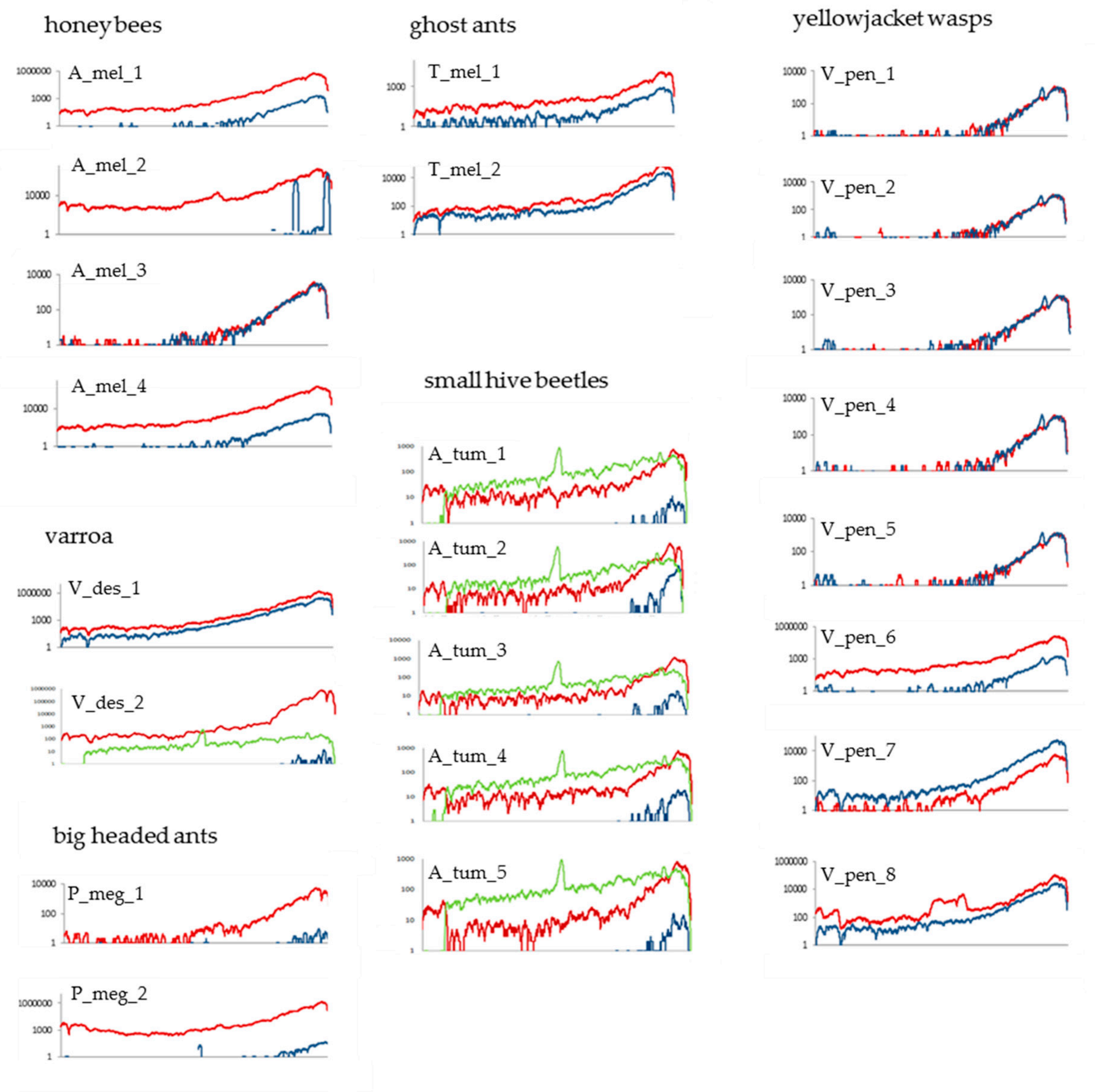

Figure S1: DWV genome coverage plots for individual samples created using Geneious. Read depths are shown on a log-10 scale and represent DWV-A (red), -B (blue), and –C (green) along the ~10.1 kb genomes.

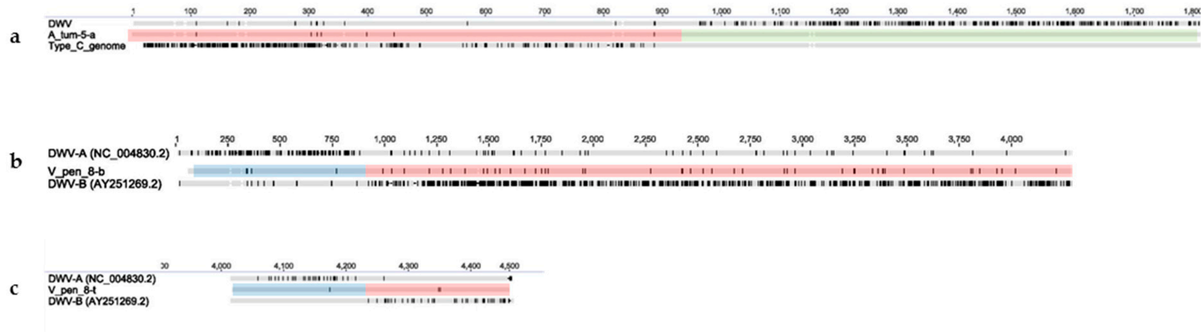

Figure S2: DWV alignments (MUSCLE) created using Geneious showing de novo assembled contigs from samples A_tum-5 and V_pen-8, which contain recombination breakpoints. (**a**) Contig A_tum-5-a aligned with DWV-A (NC_004830.2) and DWV-C (CEND01000001.1) reference genomes, (**b**) contig V_pen_8-b aligned with DWV-A (NC_004830.2) and DWV-B (AY251269.2), and (c) a second contig from sample V_pen_8; V_pen_8-t also aligned with DWV-A (NC_004830.2) and DWV-B (AY251269.2). All alignments show disagreements with the consensus sequences highlighted in black and recombinant contigs are shaded red where they map most closely to DWV-A, blue to DWV-B, and green to DWV-C.

Table S1: Samples used in this study. Sample names are given along with the site from which they were sampled, species name, and the symbol used to denote them in Figures 1 and 5.

Sample	Site	Species	Symbol
T_mel_1	B2	*T. melanocephalum*	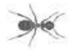
T_mel_2	B2	*T. melanocephalum*	
P_meg_1	B3	*P. megacephala*	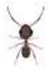
P_meg_2	O1	*P. megacephala*	
A_tum_1	O2	*A. tumida*	
A_tum_2	B1	*A. tumida*	
A_tum_3	B1	*A. tumida*	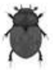
A_tum_4	B1	*A. tumida*	
A_tum_5	B1	*A. tumida*	
V_pen_1	B4	*V. pensylvanica*	
V_pen_2	B4	*V. pensylvanica*	
V_pen_3	B4	*V. pensylvanica*	
V_pen_4	B4	*V. pensylvanica*	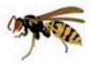
V_pen_5	B4	*V. pensylvanica*	
V_pen_6	B3	*V. pensylvanica*	
V_pen_7	B3	*V. pensylvanica*	
V_pen_8	B3	*V. pensylvanica*	
A_mel_1	B3	*A. mellifera*	
A_mel_2	O1	*A. mellifera*	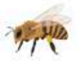
A_mel_3	B1	*A. mellifera*	
A_mel_4	B2	*A. mellifera*	
V_des_1	B3	*V. destructor*	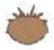
V_des_2	O1	*V. destructor*	

Table S2: DWV-A *RdRp* sequences originally from [4] and used in this study in the construction of the DWV-A phylogeny in Figure 5.

Accession no	host	country	year
KP734679	*A. mellifera*	France	2009
KP734616	*A. mellifera*	Germany	2009
KP734641	*A. mellifera*	Hawaii	2009
KP734623	*V. destructor*	Hawaii	2009
KP734625	*V. destructor*	Hawaii	2009
KP734687	*A. mellifera*	Hawaii	2009
KP734692	*A. mellifera*	Pakistan	1998
KP734713	*V. destructor*	Poland	2009
KP734653	*A. mellifera*	UK	2009
KP734694	*A. mellifera*	USA	2009

Table S3: Viruses commonly found in bees used for BLAST analysis along with accession numbers.

Virus	Accession number
Deformed wing virus – type A	NC_004830.2
Deformed wing virus – type A, Kakugo virus	NC_005876.1
Deformed wing virus – type B, Varroa destructor virus 1	AY251269.2
Deformed wing virus – type C	ERS657949
Milolii virus	MF155030.1
Moku virus	NC_031338.1
Acute bee paralysis virus (ABPV)	NC_002548.1
Black queen cell virus (BQCV)	NC_003784.1
Israeli acute paralysis virus (IAPV)	NC_009025.1
Kashmir bee virus (KBV)	NC_004807.1
Lake Sinai virus (LSV)	NC_032433.1
Sacbrood virus (SBV)	NC_002066.1
Slow bee paralysis virus (SBPV)	NC_014137.1

Table S4: Numbers of reads mapping to DWV types A, B, and C using BLAST top hit analysis for each sample, along with the total numbers of reads passing QC (read1.fasta).

Sample	DWV A	DWV B	DWV C	Total DWV	Total reads
T_mel_1	92713	11747	461	104921	19669974
T_mel_2	536208	183603	1009	720820	17461610
P_meg_1	47074	3128	844	51046	18268406
P_meg_2	8984109	691892	356	9676357	19795762
A_tum_1	6636	808	15661	23105	18581324
A_tum_2	6713	1031	9758	17502	16891197
A_tum_3	9594	941	9493	20028	16609397
A_tum_4	6612	898	14267	21777	17083341
A_tum_5	7027	772	17290	25089	17683443
V_pen_1	8757	9670	178	18605	11961813
V_pen_2	10718	11858	82	22658	12807830
V_pen_3	9709	11883	144	21736	14181324
V_pen_4	9735	15421	48	25204	16089890
V_pen_5	10981	13184	81	24246	17001465
V_pen_6	956354	18679	717	975750	15504637
V_pen_7	37106	371054	156	408316	14215385
V_pen_8	687229	242371	586	930186	13394271
A_mel_1	3839027	141563	239	3980829	17678449
A_mel_2	4877249	269833	7187	5154269	13765791
A_mel_3	24613	24797	241	49651	12183541
A_mel_4	14705870	512418	131	15218419	23505172
V_des_1	9653918	2452020	71	12106009	13498757
V_des_2	28031169	2089009	1876468	31996646	35064635
